# A visible seedling‐stage screening system for the *Brassica napus* hybrid breeding by a novel hypocotyl length‐regulated gene *BnHL*

**DOI:** 10.1111/pbi.14507

**Published:** 2024-11-06

**Authors:** Jingyan Fu, Ying Zhang, Meng Yin, Sha Liu, Ziyue Xu, Mingting Wu, Zihan Ni, Peiyao Li, Ruijia Zhu, Guangqin Cai, Maolin Wang, Rui Wang

**Affiliations:** ^1^ Key Laboratory for Bio‐Resources and Eco‐Environment, College of Life Sciences Sichuan University Chengdu China; ^2^ Oil Crops Research Institute Chinese Academy of Agricultural Sciences Wuhan China

**Keywords:** *Brassica napus*, genic male sterility, hybrid seed production, hypocotyl elongation, gene editing

## Abstract

Rapeseed (*Brassica napus*) is a globally significant oilseed crop with strong heterosis performance. Recessive genic male sterility (RGMS) is one of the key approaches for utilizing heterosis in *B. napus*. However, this method faces the inherent challenge of being time‐consuming and labour‐intensive for removing fertile plants during seed production. Here, we report a hypocotyl length‐regulated gene, *BnHL*, which is closely linked to a known fertility gene, *BnMs2*, serving as a seedling morphology marker. This marker could be used to identify fertile plants in the breeding of RGMS lines based on hypocotyl traits. By targeting the *BnHL* gene, both homozygous and heterozygous edited mutants exhibited significantly longer hypocotyls than the wild type (WT). Furthermore, germination experiments revealed that 7 days after seed germination, the difference in hypocotyl length between the mutant and the WT seedlings reached its maximum, effectively distinguishing fertile plants under both white (W) and red/far‐red (R/FR) light. Mutations in *BnHL* did not result in significant changes in main agronomic traits. Thus, this study provides a comprehensive strategy for screening and identifying a new morphological marker gene for early screening in RGMS hybrid breeding with completely non‐transgene during the whole production.

## Introduction

Heterosis refers to the phenomenon where hybrids exhibit superior agricultural and comprehensive traits compared to their parent line (Birchler, 2015; Coors and Pandey, 1999; Shull, 1948). It has been extensively studied and applied to key crops such as rice (*Oryza sativa*), maize (*Zea mays*), soybean (*Glycine max*) and rapeseed (*Brassica napus*) (Bohra *et al*., 2016; Huang *et al*., 2016; Wang *et al*., 2023a, 2023b; Ye *et al*., 2023). Rapeseed shows significant heterosis, and studies on hybrid breeding have greatly improved the quality and yield of rapeseed (Fu, 2000; Gehringer *et al*., 2007). Currently, two main approaches are used to utilize heterosis in rapeseed: cytoplasmic male sterility and genic male sterility (GMS). However, both systems possess inherent limitations (Chang *et al*., 2016).

Recessive genic male sterility (RGMS) system has been widely used due to its stable fertility, lack of negative cytoplasmic effects and broad recovery range (Fu and Zhou, 2013). RGMS lines include S45A and 117A, among others. Male sterility is controlled by two duplicate recessive genes, *Bnms1* and *Bnms2* (Hou *et al*., 1990; Pan *et al*., 1988). The genotypes of the sterile (A line) and fertile (B line) plants are *Bnms1ms1ms2ms2* and *BnMs1ms1ms2ms2* respectively (Yi *et al*., 2006). However, when recessive genes are used for hybrid seed production, crosses between homozygous sterile lines and heterozygous fertile maintainer lines yield sterile‐to‐fertile plants in a 1:1 ratio. Consequently, it is challenging to efficiently eliminate fertile plants over large areas when utilizing recessive sterility for seed production. Positional cloning of male‐sterile genes enables precise selection of sterile plants using molecular markers, but this approach increases costs and technical complexity. Furthermore, attempts have been made to use morphological markers closely linked to relevant genes for early‐stage selection to distinguish sterile genotypes (Francsco *et al*., 2005; Gardner, 2000; Tanksley *et al*., 1984).

In recent years, the continuous refinement of gene editing systems has prompted scientists to utilize CRISPR/Cas9 technology to generate male‐sterile lines across various species. The integration of T‐DNA containing fertility and marker genes into these sterile lines enables the development of maintainer lines, effectively addressing challenges related to sterile plant selection. These marker genes could cause visible phenotype, such as fluorescent gene that causes maize kernels in maintainer lines to emit red fluorescence (Qi *et al*., 2020), anthocyanin synthesis regulated gene that results in purple seedlings of the maintainer lines (Du *et al*., 2020), and anthocyanin accumulation defective gene that generates green hypocotyls (Zhou *et al*., 2023), etc. However, this approach involves the large‐scale introduction of large DNA fragments into the host plant genome. The genetic stability of sterility genes and the effects of large fragments on other agronomic traits have not yet been explored.

In the present study, we proposed a comprehensive strategy for *de novo* screening and identification of a new morphological marker gene for a visible seedling‐stage screening (VSS) system. First, a novel seedling‐stage hypocotyl length‐regulated gene was screened and functionally characterized, which could be applied for the early identification of sterile plants. Its application feasibility has been further confirmed in *B. napus* by a co‐segregation test. Germination experiments indicate that the 7th day of hypocotyl growth is the optimal period for distinguishing fertile plants. This system shows significant potential and avoids the unexpected effects of transgenic on plants (Ladics *et al*., 2015; Schnell *et al*., 2015), thereby facilitating the application of the GMS system and enriching the utilization of heterosis, which could be expected to bring a breakthrough in the breeding of new hybrid rapeseed varieties.

## Results

### Seedling‐stage visible marker screening strategy for sterile plants selection

**Figure 1:**
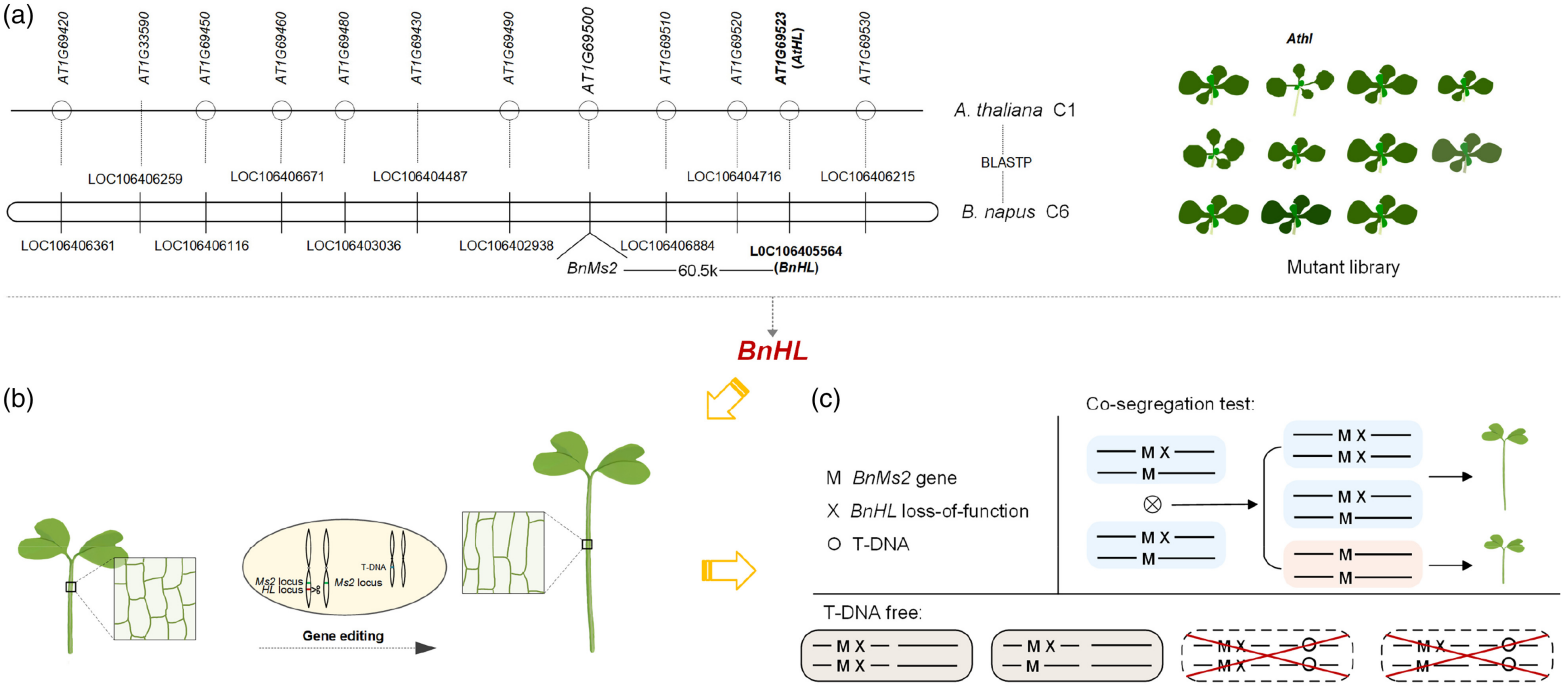
Strategy for *de novo* screening and identification of the new candidate gene as non‐transgenic marker in plant breeding. (a) Candidate genes closely linked to *BnMs2* and corresponding homologous *Arabidopsis* gene mutant library. (b) CRISPR/Cas9 technology was performed on the *B. napus*. The patterns in the large square represent the hypocotyl cells of the plant respectively. Scissors indicate editing of the locus. (c) A schematic for the co‐segregation test and T‐DNA free lines screening.

Twenty candidate genes closely linked to *BnMs2* were selected from the 100 kb region surrounding *BnMs2*. Based on the identities of *B. napus* and *A. thaliana*, 11 homologous genes in *A. thaliana* with a sequence coverage of over 90% and similarity of over 70% were selected (Table S1). Corresponding *Arabidopsis* mutants were purchased from The Arabidopsis Information Resource (TAIR) and confirmed to be homozygous mutants. These mutants were then planted alongside the wild type (WT, Col‐0) plants to observe their phenotypic characteristics throughout the growth cycle (Figure S1). Within the *Arabidopsis* mutant library, an *Athl* mutant with hypocotyl elongation was screened, and the corresponding homologous gene in *B. napus*, *BnHL*, was selected as the marker gene (Figure 1a). Gene editing targeting *BnHL* was conducted to identify its phenotype in *B. napus* (Figure 1b). Subsequently, a co‐segregation test and screening of T‐DNA‐free lines were performed with phenotypic investigation and PCR identification to generate the non‐transgenic GMS marker (Figure 1c).

### *Athl* mutant phenotype analysis

**Figure 2:**
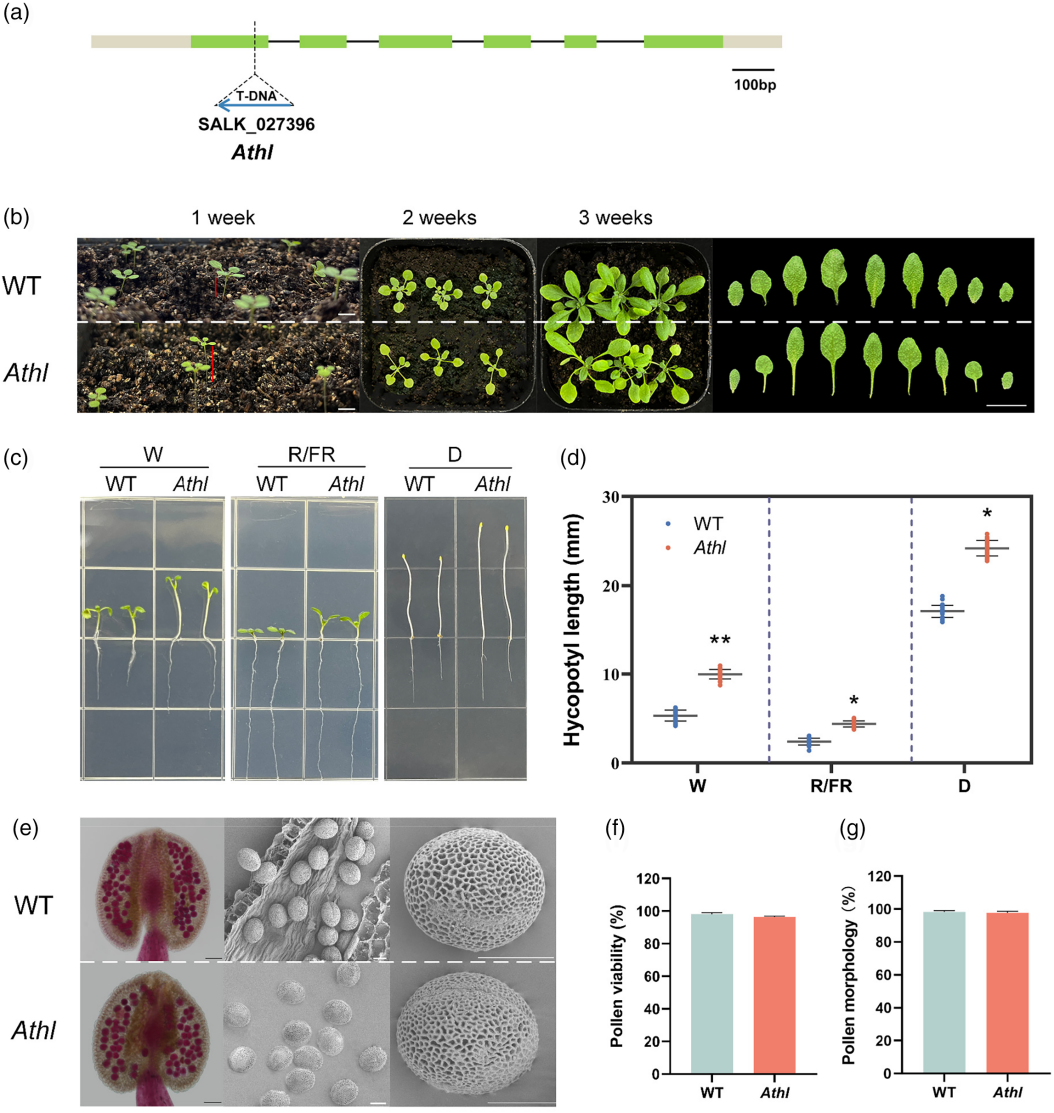
Phenotypes of *Arabidopsis* wild‐type (WT) and *Athl* mutant. (a) Structures of *Athl* and T‐DNA insertion in the first exon. Bar = 100 bp. (b) Comparison of WT and *Athl* mutant in the 1st, 2nd and 3rd weeks of growth, and differences in petiole. The red line indicates the hypocotyl length at 1 week. Bar = 1 cm. (c, d) Hypocotyl length of WT and *Athl* under white light (W), red/far‐red light (R/FR) and dark (D) respectively. 15 mm grid edge length (*P < 0.05, **P < 0.01, three independent repeats, *n* = 30). (e) Alexander staining to detect the viability of mature pollen and morphology of mature pollen grains observed by SEM. From left to right, bars = 10, 100 and 10 μm. (f, g) Percentage of pollen viability by Alexander staining and normal morphology by SEM (three independent repeats, *n* = 60).

In the *Arabidopsis* mutant library, the T‐DNA insertion mutant SALK027396 of *AT1G69523*, which displayed a phenotype distinct from that of the WT, was named *Athl* (hypocotyl length). Specific primers of the *AtHL* gene (ATHL‐LP and ATHL‐RP) and universal primers of T‐DNA (LBb1.3) were used for PCR amplification (Figure S2), and the amplified products were sequenced. Sequence analysis revealed that the T‐DNA of the *Athl* was inserted into the first exon of *AT1G69523* (Figure 2a). Observations of *Athl* revealed longer hypocotyls, smaller leaf volume, lighter‐coloured leaves, and longer petioles compared to the WT (Figure 2b). Based on the phenotype shown in Figure 2b, both WT and *Athl* seeds were plated on 1/2MS medium to further investigate the differences in hypocotyl length (Figure 2c). It has been reported that red and far‐red light inhibit hypocotyl elongation (Lau and Deng, 2010; McNellis and Deng, 1995). Germination experiments were conducted under white (W) light, red/far‐red (R/FR) light, and dark (D) conditions to exclude the potential effects of different light qualities on this phenotype. Subsequent measurements of hypocotyl length showed that the hypocotyl length of *Athl* (10.21 ± 0.56 mm) significantly exceeded that of the WT (5.54 ± 1.06 mm) under W light. Similarly, the hypocotyl length of *Athl* (4.39 ± 0.33 mm) was significantly longer than that of the WT (2.41 ± 0.38 mm) under R/FR light. In the dark environment, both *Athl* and WT exhibited considerable increases in hypocotyl length compared to the other two light conditions; however, the hypocotyl length of *Athl* (24.20 ± 0.85 mm) remained significantly longer than WT (17.11 ± 0.68 mm) (Figure 2d). These findings consistently demonstrated that under different light qualities, the hypocotyl length of the *Athl* surpassed WT, indicating a stable phenotype.

The effects of mutations in this gene on plant fertility were also investigated. Alexander staining results indicated normal cytoplasmic morphology of pollen in *Athl*, with no significant difference from the WT (Figure 2e,f). Additionally, scanning electron microscopy (SEM) examination of mature pollen walls showed that there is no significant difference in the outer wall morphogenesis between *Athl* and WT (Figure 2e,g).

### Function of *BnHL* in regulation of hypocotyl length

**Figure 3:**
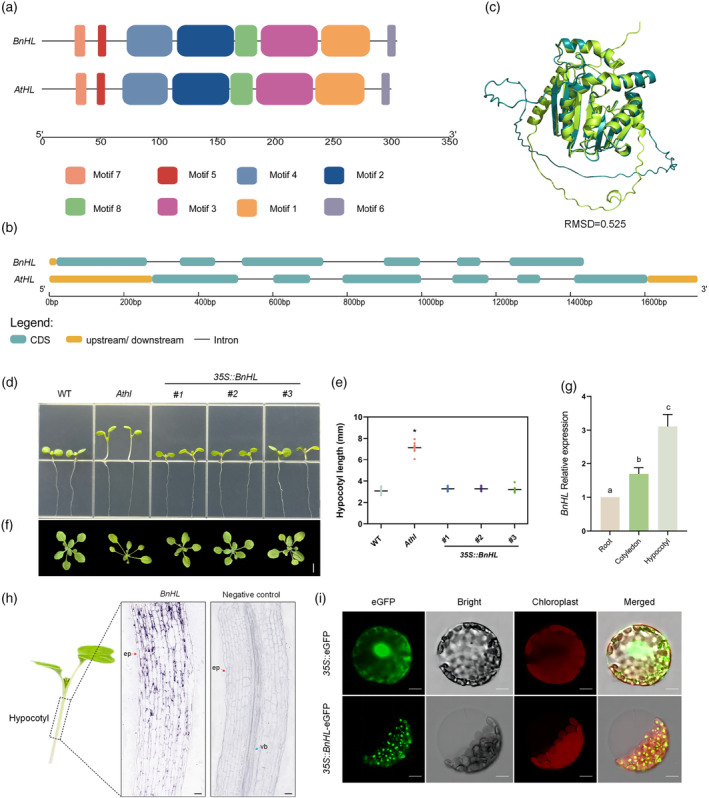
Exploration of the function of *BnHL* in regulating hypocotyl length. (a) Conserved primary structural feature (motifs) of *BnHL*. Different coloured squares indicate different motifs. (b) Gene structure of *BnHL*. (c) Predicted 3D structure of the BnHL protein. (d, e) Hypocotyl length comparison of wild type (WT), *Athl* and transgenic *Arabidopsis* plants overexpression *BnHL* in *Athl* mutant. (*P < 0.05, three independent repeats, *n* = 10). 15 mm grid edge length. (f) Morphological comparison of WT, *Athl* and transgenic *Arabidopsis* plants overexpression *BnHL* in *Athl* mutant. Bar = 1 cm. (g) Relative *BnHL* transcript levels in the root, cotyledon, and hypocotyl of *B. napus* seedlings. Three independent repeats, different lowercase letters indicate significant differences (*P* < 0.05). (h) *In situ* hybridization of *BnHL* messenger RNA (mRNA) in the hypocotyl of 7‐day‐old *B. napus* seedlings. Red arrowheads indicate the epidermis (ep), and blue arrowheads indicate vascular bundles (vb). Bars = 100 μm. (i) Subcellular localization of *BnHL* in *N. benthamiana* protoplasts by fusion eGFP. Bars = 30 μm.

The mutation of *AT1G69523* leads to an elongated hypocotyl phenotype, indicating that *AtHL* may be involved in the regulation of hypocotyl length. Consequently, the corresponding homologous gene in *B. napus*, LOC106405564 (named *BnHL*), was also investigated. *BnHL* shares 73.75% amino acid sequence similarity with *AtHL* (Table S1), and both belong to the methyltransferase superfamily. Initially, an online motif analysis of *BnHL* and *AtHL* was conducted using MEME, and then visualized using TBtools. The same eight conserved motifs with consistent distribution and high homology were predicted (Figure 3a). Gene structure visualization using GSDS2 revealed similar architectures for both genes (Figure 3b). Finally, a 3D spatial structure prediction of the translated proteins was performed with SWISSMODEL, followed by alignment comparison using PyMOL to calculate root‐mean‐square deviation (RMSD) values, yielding an RMSD of 0.525 between the BnHL and AtHL proteins, signifying significant structural similarity (Figure 3c).

A complementation assay was conducted to validate the role of *BnHL* in the regulation of hypocotyl length. The results revealed that transgenic lines expressing *BnHL* exhibited a normal WT hypocotyl phenotype (Figure 3d). Hypocotyl lengths in transgenic lines #1, #2 and #3 did not significantly differ from those in the WT but showed significant differences compared to that of *Athl* (Figure 3e). Additionally, the rosette phenotype of the transgenic lines was restored to WT‐like characteristics (Figure 3f). Subsequently, the expression levels of *BnHL* in the tissues of 7‐day‐old seedlings were analysed. The transcript level of *BnHL* was relatively low in the cotyledons and roots but notably abundant in the hypocotyls (Figure 3g). Furthermore, *in situ* hybridization was employed to validate the spatial expression pattern of *BnHL* in the hypocotyls (Figure 3h). The *BnHL* transcript signal (purple colour) showed higher expression in the upper region of the hypocotyl, with discernible but lower expression in the lower region. Transient expression analysis demonstrated that the *BnHL*‐eGFP fusion protein exhibited punctate aggregation in chloroplasts (Figure 3i). These results further confirm the role of *BnHL* in the regulation of hypocotyl length in *B. napus*.

### Targeted mutations in *BnHL* lead to seedling hypocotyl elongation

**Figure 4:**
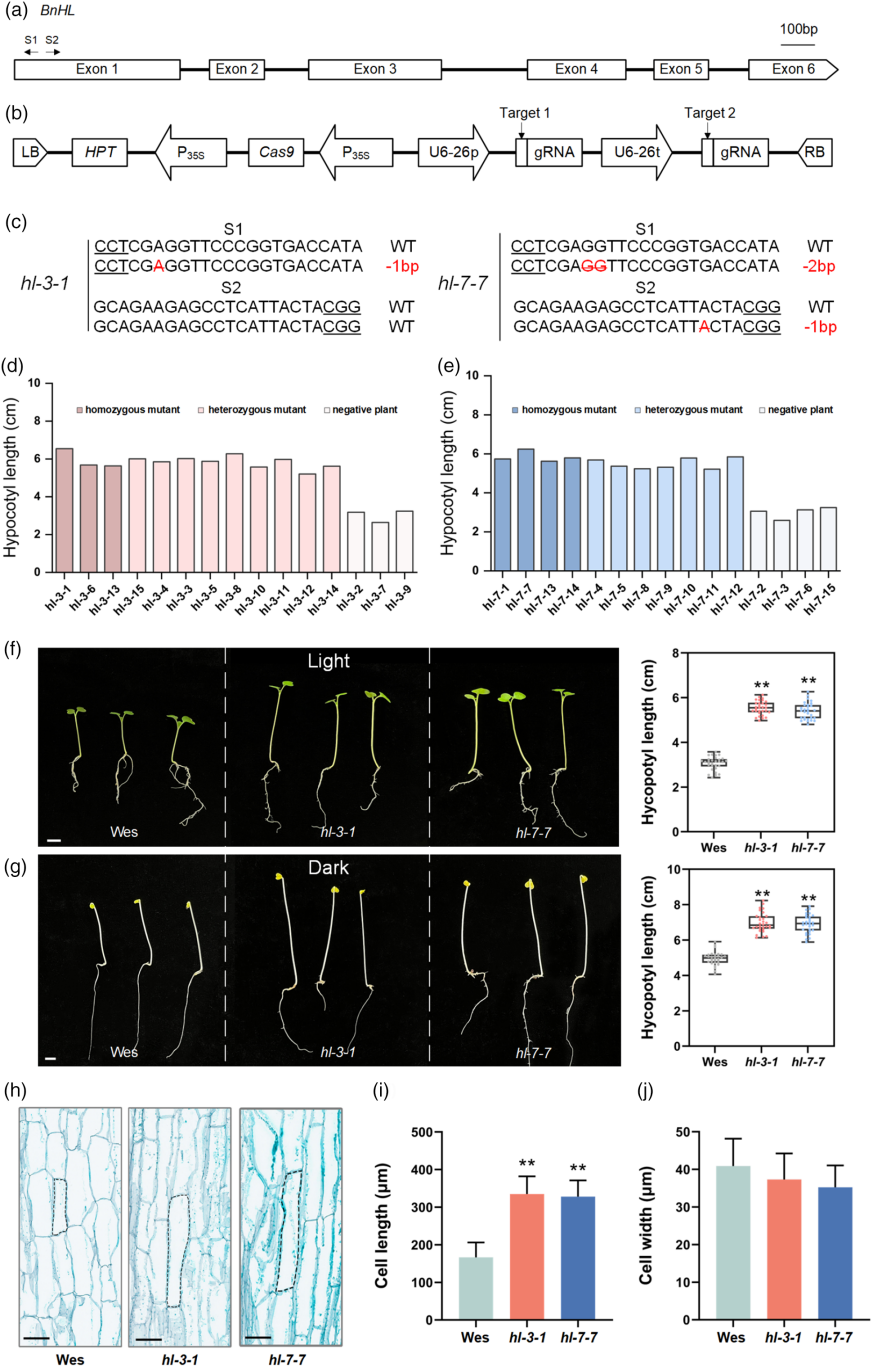
Phenotype of *Bnhl* mutant in *B. napus*. (a) The *BnHL* gene model includes six exons (box) separated by five introns (solid line). The arrow indicates the sgRNA direction. (b) Vector structure of the CRISPR/Cas9 hosting two sgRNA expression cassettes. The vertical arrow in the gene model indicates the target site. (c) Sequencing at the mutation sites of the *Bnhl* in the T_1_ generation. The PAM is underlined, and nucleotide deletions are marked in red, with details labelled at the right. (d‐e) Hypocotyl length of 15 T_I_ generation plants of *hl‐3* and *hl‐7*. (f, g) Hypocotyl length of Wes and *Bnhl* mutants in light and dark respectively. *hl‐3‐1* and *hl‐7‐7* in the figure represent their offspring respectively (**P < 0.01, three independent repeats, *n* = 30). Bars = 1 cm. (h, j) Cell length (i) and width (j) of 7‐day‐old Wes and *Bnhl* mutant seedling hypocotyls (**P < 0.01, three independent repeats, *n* = 24). Bars = 100 μm.

To generate mutations in *BnHL* using the CRISPR/Cas9 system, two sgRNAs, sgRNA (S1) and sgRNA (S2), were designed to target the first exon of *BnHL* to ensure a successful knockout (Figure 4a), and Cas9 was driven by the 35S promoter (Figure 4b). Positive T_0_ transgenic lines were identified by PCR. Among the positive T_0_ transgenic plants, two *Bnhl* mutants were identified, both exhibiting deletions at the target sites within *BnHL*, resulting in frameshift mutations (Figure 4c). To obtain stable mutant lines, two individual T_0_ editing lines of *Bnhl* were self‐pollinated to generate T_1_ progeny lines. Fifteen randomly selected T_1_ progenies were sequenced, and their hypocotyl lengths were measured (Figure 4d,e). The results revealed that both heterozygous and homozygous mutants exhibited longer hypocotyls than negative plants (plants without editing). Two distinct homozygous double mutant lines (*hl‐3‐1* and *hl‐7‐7*) were selected for further analysis.

On the 7th day post‐germination under normal growth conditions, the emergence of the first true leaf signalled the cessation of hypocotyl growth (Chen *et al*., 1978). Seeds from Westar (Wes) and *Bnhl* mutants (*hl‐3‐1* and *hl‐7‐7*) were vernalized and germinated in Petri dishes under normal white light and complete darkness, respectively, and hypocotyl lengths were measured after 7 days. Under normal light, the hypocotyl length of both *hl‐3‐1* (5.54 ± 0.39 cm) and *hl‐7‐7* (5.39 ± 0.38 cm) were significantly longer than Wes (3.06 ± 0.30 cm) (Figure 4f). In a dark environment, *Bnhl* mutants and Wes showed skotomorphogenesis phenotypes, that is, slender and long hypocotyls with curved hook‐like bends in the apical leaves, as well as small, yellow leaves. The hypocotyl length of *hl‐3‐1* (6.98 ± 0.55 cm) and *hl‐7‐7* (6.92 ± 0.53 cm) were also significantly longer than Wes (4.95 ± 0.39 cm) (Figure 4g). To investigate the histological differences in hypocotyl length between Wes and *Bnhl* mutants, the length and width of slitting cells in 7‐day‐old hypocotyls grown under normal light conditions were measured. Compared to Wes, the cell lengths of *hl‐3‐1* and *hl‐7‐7* approximately doubled, while no significant difference in cell width was observed (Figure 4h–j). These results suggest that the elongation of the hypocotyl in *Bnhl* mutants is mainly controlled by longitudinal cell elongation, which is consistent with existing reports (Gendreau *et al*., 1997; Kutschera and Niklas, 2007).

### Feasibility validation of the seedling‐stage visible marker in GMS line

**Figure 5:**
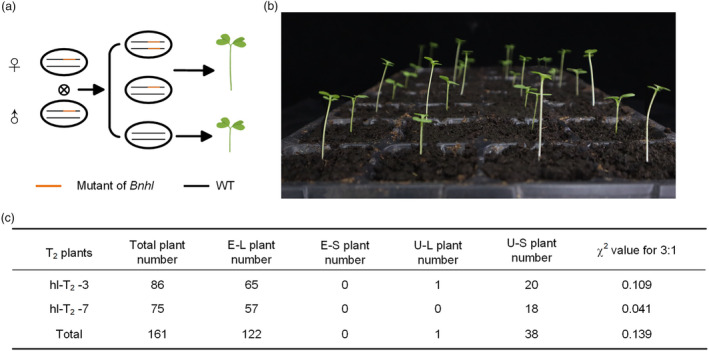
Co‐segregation test of the *Bnhl*. (a) A schematic for the co‐segregation test. (b) Representative image of co‐segregation by self‐pollination. (c) The identification of T_2_ plants by hypocotyl phenotypes and sequence results. E–L indicates edited plants with long hypocotyls; E‐S indicates edited plants with short hypocotyls; U–L indicates unedited plants with long hypocotyls; and U–S indicates unedited plants with short hypocotyls. The one‐sided χ^2^ test for 3:1 of E–L plants to U–S plants were conducted with significance level at 0.05. χ^2^_0.05,1_ = 3.84. T_2_, transgenic generation 2.

Phenotypic and genotypic analyses of the T_1_ generation revealed that all homozygous and heterozygous plants edited for *BnHL* showed elongated hypocotyls, while the unedited plants did not exhibit this phenotype, indicating that *Bnhl* co‐segregated with the phenotype of hypocotyl elongation. To further confirm this result, the lines *hl‐3‐8* and *hl‐7‐12* were selected. The progenies were generated through self‐pollination to produce segregated populations for the *Bnhl* locus (hl‐T_2_‐3 and hl‐T_2_‐7; Figure 5a). Based on previous experimental data, a hypocotyl length of 4 cm was initially established as the standard, plants with hypocotyl lengths exceeding 4 cm were classified as having long hypocotyls, and those below 4 cm were classified as having short hypocotyls (Figure 5b). Phenotypic analysis of individuals from the S_1_ population indicated that among the 86 descendants generated in hl‐T_2_‐3, there were 65 edited plants with long hypocotyls, 20 unedited plants with short hypocotyls, and one unedited plant that appeared to have a longer hypocotyl. Among the 75 descendants generated in hl‐T_2_‐7, there were 57 edited plants with long hypocotyls and 18 unedited plants with short hypocotyls. A χ^2^ test showed that the ratio of edited plants with long hypocotyls to unedited plants with short hypocotyls matched the expected 3:1 segregation ratio (hl‐T_2_‐3: χ^2^_0.05_ = 0.109 < 3.84; hl‐T_2_‐7: χ^2^_0.05_ = 0.041 < 3.84; Figure 5c), thereby confirming the feasibility and stability of hypocotyl length as a VSS marker.

### Optimal timing for the removal of fertile plants

**Figure 6:**
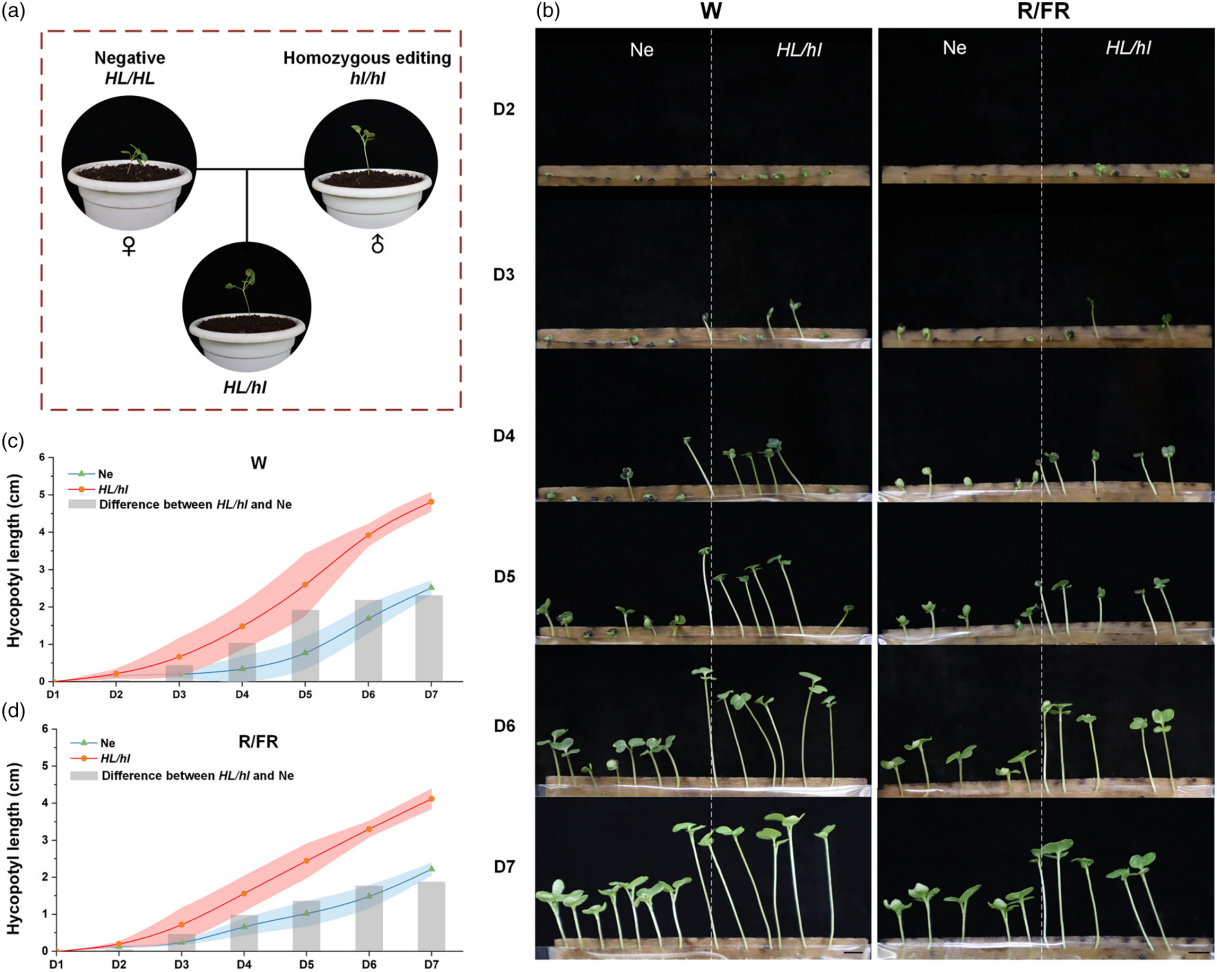
Trends in hypocotyl growth between heterozygous edited plants (*HL/hl*) and negative plants (Ne). (a) Hybridization schematic. (b–d) Hypocotyl length changes from D2 to D7 of Ne and *HL/hl* under white (W) and red/far red (R/FR) light respectively (three independent repeats, *n* = 7). Bars = 1 cm.

To obtain heterozygous edited plants for further exploration of the application potential of this marker and its optimal sorting time, homozygous edited plants from the T_2_ generation were crossed with negative plants (Ne) (Figure 6a). Germination experiments on the resulting heterozygous edited plants and Ne were then performed to observe the period during which the greatest differences in hypocotyl length occurred between the two plant groups. Given the complex qualities of natural light, W and R/FR light conditions were established for germination. Sterilized seeds were sown in seed germination pouches with seven plants each of *HL/hl* and Ne per pouch (noting that some loss occurred due to seedling wilting during growth). Hypocotyl length changes were monitored from day 2 (D2) to day 7 (D7) post‐germination. The results showed that the hypocotyl growth rate of *HL/hl* was consistently accelerated compared to Ne under both light conditions (Figure 6b). The hypocotyls under R/FR were generally shorter than those under W, however, the differences in hypocotyl lengths reached a maximum on D7 in both cases (Figure 6c,d). Therefore, the 7th day of hypocotyl growth was considered as the most appropriate time to differentiate between sterile and fertile plants for the purpose of removing the fertile ones.

### The VSS system for hybrid seed production

To determine whether editing *BnHL* affects the main agronomic traits of rapeseed, the self‐pollinating families hl‐T_3_‐3 and hl‐T_3_‐7 were selected for evaluation. The results showed that the editing lines exhibited no significant differences compared to Wes in terms of yield, 1000‐seed weight, seed numbers per silique and plant height (Table S3).

**Figure 7:**
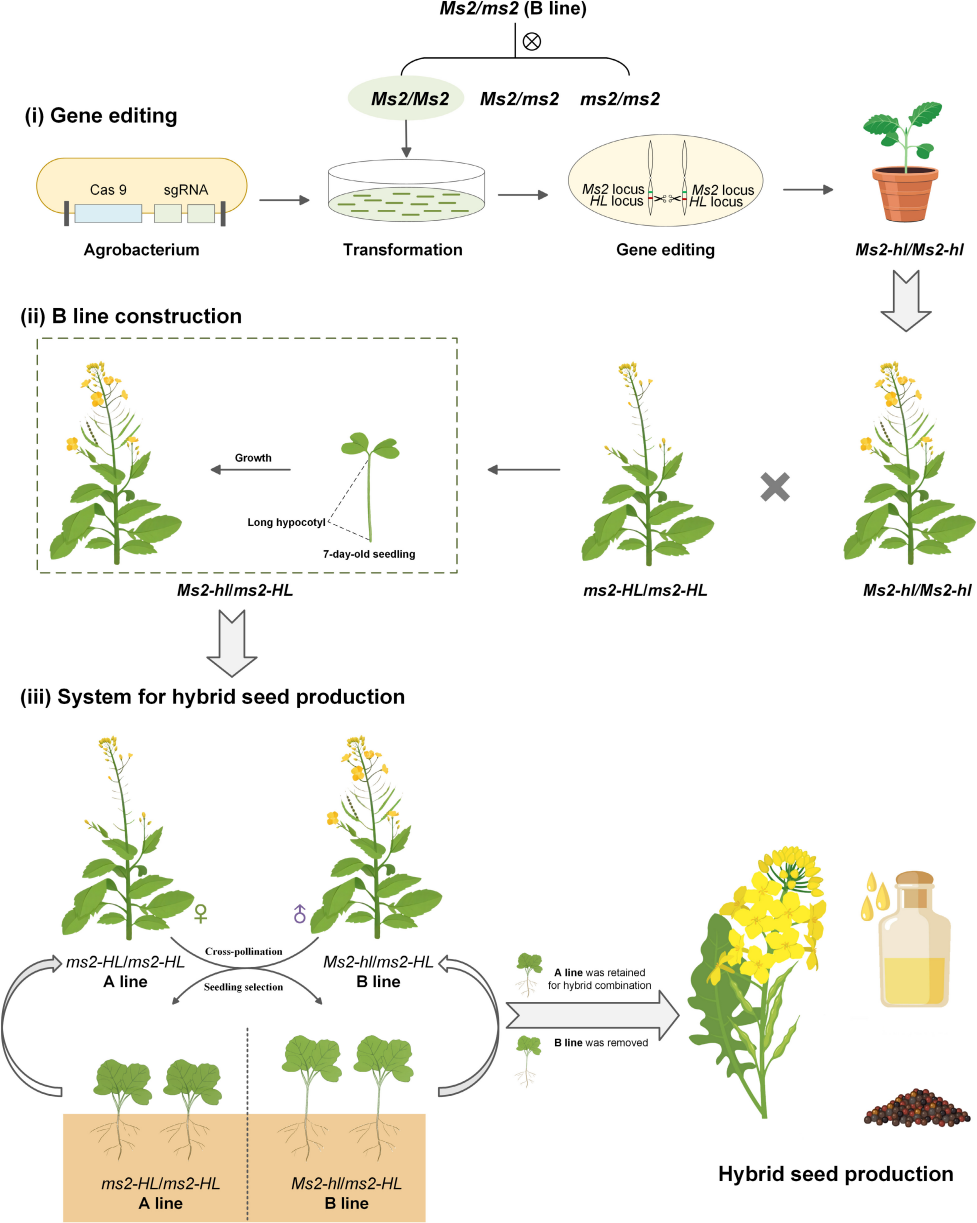
The visible seedling‐stage screening (VSS) system for hybrid production in *B. napus*. (i) Homozygous edited plants were obtained by CRISPR/Cas9‐mediated mutagenesis of *BnHL*. (ii) B line was obtained by crossing a homozygous edited plant with A line. (iii) B line and A line continue to be produced through cross‐pollination, and non‐transgenic male‐sterile plants are easy to identify and select for hybridization.

The above results suggested that this system has a great potential for hybrid breeding application. The entire process consists of three steps: (i) First, the B line (*Ms2/ms2*, all material genotypes were *ms1/ms1* with no additional information indicated) in two‐line was self‐pollinated to obtain materials with the genotype *Ms2/Ms2*. Editing *BnHL*, which is closely linked to the fertility gene *Ms2*. (ii) Hybridizing the homozygous *Bnhl* mutant (genotype: *Ms2‐hl/Ms2‐hl*) with A line (genotype: *ms2‐HL/ms2‐HL*) to acquire the heterozygous *Bnhl* mutant (B line, genotype: *Ms2‐hl/ms2‐HL*). (iii) Cross‐pollinating the B line with the A line as the paternal parent, resulting in a 1:1 ratio of sterile plants (genotype: *ms2‐HL/ms2‐HL*) to fertile plants (genotype: *Ms2‐hl/ms2‐HL*). both of which were non‐transgenic, effectively avoiding horizontal transfer of transgenic components. Seven days after germination, fertile plants exhibited significantly elongated hypocotyls, which allowed fertile lines to be distinguished, and male‐sterile plants could be used to produce hybrid seeds (Figure 7).

## Discussion

Rapeseed shows significant heterosis, and with easy mastery of hybrid seed production, many efforts have been made to develop its hybrid breeding techniques (Tan *et al*., 2024). RGMS is currently the most widely used sterility system for exploiting heterosis in rapeseed research. However, a notable limitation of RGMS is the absence of a complete maintainer line, which necessitates a two‐line system. This approach requires the removal of all male fertile plants during flowering based on fertility assessments to generate F_1_ hybrid seeds. One strategy to address this challenge is to identify morphological markers that are closely or completely linked to male sterility at the seedling or seed stage. These markers enable the distinction between sterile and fertile plants, facilitating separate transplantation or removal. Next‐generation GMS hybrid biotechnology offers an effective solution for sorting GMS and maintainer seeds using the seed production technology (SPT) process (Wu *et al*., 2016). SPT involves introducing a normally fertile gene, a pollen lethality gene, and a fluorescent protein gene with linked expression into a common GMS line. This resulted in a 1:1 ratio of fluorescent fertile to non‐fluorescent sterile seeds, enabling selection based on seed colour. Scientists have further refined various aspects of this technology for the large‐scale reproduction of male‐sterile seeds in crops such as maize and rice (Cai *et al*., 2022; Chang *et al*., 2016; Fox *et al*., 2017; Zhang *et al*., 2017).

Seedling morphological markers are also widely utilized for their easy detection during early plant developmental stages (Haskell, 1961). For example, linking the fertility gene with the anthocyanin synthesis regulated gene allows for transferring these genes into male‐sterile lines, ultimately obtaining a purple maintainer line with restored fertility. Male‐sterile lines (non‐purple) can be easily distinguished based on seedling colour (Du *et al*., 2020). Other markers include leaf shape (Francsco *et al*., 2005), albino phenotype (Su *et al*., 2012) and hypocotyl colour (Zhou *et al*., 2023). These strategies involve constructing vectors that contain both fertility and marker genes to facilitate linkage, followed by transferring these vector fragments into receptor plants. *B. napus* is a typical allopolyploid crop with two subgenomes, A and C, which exhibit extensive gene duplication and copy number variation (Grant *et al*., 1998; Cavell *et al*., 1998; Parkin *et al*., 2002). This increases the probability of simultaneous silencing of both endogenous and exogenous genes when introducing foreign genes. In the present study, the CRISPR/Cas9 system was utilized to knock out the *BnHL*, which is closely linked to the fertility gene *BnMs2*. Knocking out *BnHL* results in hypocotyl elongation, serving as a visible marker that ensures trait expression while minimizing the risks of failure and unnecessary side effects associated with transferring large gene fragments into receptor plants, making it a more ‘natural’ system. The co‐segregation test and T‐DNA‐free plant screening were conducted to confirm the co‐segregation of the edited *BnHL* with hypocotyl elongation, further validating the feasibility and non‐transgenic nature of the system. In the co‐segregation test, an Ne plant exhibiting elongated hypocotyls was identified, likely due to its shaded growth environment, which lacked adequate light exposure. Hence, this system possesses more significant potential applications in mechanization and intelligent agriculture.

Male sterile and fertile plants of the same variety showed similar external morphological traits. However, they can be easily distinguished by observing characteristics such as anther shape, colour, presence or absence of pollen, stamen length and petal size. Consequently, fertile plants must be identified post‐flowering, which limits optimal land utilization for seed production. The removal of fertile plants at a later stage is more challenging and inefficient, leading to increased resource use (labour, fertilizers, water and pesticides). The hypocotyl plays a crucial role during the seedling stage by pushing the cotyledons above the soil surface for photosynthesis. Notably, previous studies have indicated that the upper quarter of the hypocotyl is a zone of rapid growth, exhibiting the highest cell elongation at any time point (Crowell *et al*., 2011). Hypocotyl elongation is influenced by environmental factors such as darkness, and internal hormone signals like indole acetic acid and brassinosteroid (Collett *et al*., 2000; Gray *et al*., 1998; Leyser *et al*., 1993; Rouse *et al*., 1998; Vandenbussche *et al*., 2005). Moreover, hypocotyl growth is driven by controlled cell expansion and heavily influenced by light (Boron and Vissenberg, 2014). In Arabidopsis, hypocotyl elongation is also associated with photoperiod. A previous study identified putative homologues of human methyltransferase‐like 16 (METTL16) and *FIONA1* (*AT2G21070*) in *Arabidopsis* (Wang *et al*., 2022). *FIONA1* is a positive regulator of photomorphogenesis, and the *FIONA1* mutant (*fio1‐1*) leads to early elongation of hypocotyls in a photoperiod‐dependent manner (Kim *et al*., 2008). *BnHL* is a putative methyltransferase‐like protein 7A (METTL7A) that belongs to the same methyltransferase superfamily as METTL16. Additionally, our results suggest that *BnHL* is localized in chloroplasts. Based on this, it was speculated that *BnHL* might be associated with light exposure, thereby influencing hypocotyl length; however, the specific mechanisms remain to be elucidated. Upon gene editing *BnHL*, heterozygous mutants exhibit phenotypes resembling those of homozygous mutants, potentially due to the dominant negative effects commonly observed in allopolyploids. Many phenotypic differences of allopolyploid hybrids could be attributed to the dominant negative effects caused by complex polymers formed from alleles of different derivation (Adams *et al*., 2003; Xin *et al*., 2020; Zhao *et al*., 2024). In RGMS hybrid breeding, it is necessary to continuously cross two‐line to generate male‐sterile lines. Therefore, the phenotype of heterozygote can be effectively utilized in male‐sterile plants screening. Previous studies showed that whether the cultivation environment is dark or light, all hypocotyl cells reach their maximum growth rate on the 3rd to 5th day after germination, plateauing after the 6th day (Crowell *et al*., 2011; Gendreau *et al*., 1997; Refrégier *et al*., 2004). Our finding is in accordance with this pattern, identifying the 7th day as optimal for discerning the maximum hypocotyl length differences between fertile and sterile plants. This stage facilitates the efficient removal of fertile plants and minimizes resource and labour inputs.

In this study, we identified a novel gene regulating hypocotyl length to construct a VSS system for distinguishing male‐sterile plants. In this system, both fertile and sterile plants are non‐transgenic during hybrid seed production, which completely prevents the horizontal transfer of transgenic components. This system shows great potential for two‐line applications and sterile plant sorting of *B. napus*. The present study provides further insights into the application of morphological markers and exploitation of heterosis in a broader range of plants.

## Materials and methods

### Plant materials and growth conditions

The *A. thaliana* Columbia‐0 (WT) line was used in this study, and all *Arabidopsis* mutants were purchased from TAIR (https://www.arabidopsis.org/). We used ‘Westar’ (Wes) as the *B. napus* L. transformation receptor. The seeds were obtained from Key Laboratory of Bio‐Resource and Eco‐Environment of Ministry of Education, Chengdu, China. WT and transgenic lines of *A. thaliana* and *B. napus* plants were grown in a greenhouse (16/8 h of light/dark at 22 °C).

### Genetic characterization

All *Arabidopsis* mutants used in this study were T‐DNA insertion mutants. The primers used to identify the T‐DNA insertion mutants are searchable from the website (http://signal.salk.edu/tdnaprimers.2.html) based on the mutant number. Genotypic identification of the mutants can be performed using three primer methods. Primers used are listed in Table S2.

### Hypocotyl length statistics

The *Arabidopsis* seeds were sterilized with 5% (v/v) NaCIO and vernalized at 4 °C for 2 days in the dark. Seeds were then plated on half‐strength Murashige and Skoog (1/2 MS) medium (1% sucrose, 0.75% agar, pH 5.7) and grown under W (424–724 nm, 459 nm), R/RF light (606–657 nm, 635 nm; 725–735 nm, 730 nm), or in the dark. The light conditions we used consisted of a mixture of R and FR light, produced by LED tubes. The hypocotyl length was measured using ImageJ software (http://rsb.info. nih.gov/ij) after 7 days of growth (Abràmoff *et al*., 2004; Corrales *et al*., 2014).

Sterilized *B. napus* seeds were spotted into seed germination pouches with appropriate amounts of Hoagland nutrient solution to keep the kraft paper inside the bags moist. The bags were maintained at 4 °C for 2 days for vernalization, and then grown in W and R/RF light to observe the length of the hypocotyls daily until the 7th day. The data were measured and statistically processed using ImageJ software.

### Staining and microscopy

Petals and pistils were removed from freshly dewy blooms using forceps. The pollen material was placed on a slide, to which two to three drops of Alexander staining solution were added to the pollen, which was thoroughly mixed, and immediately covered with a coverslip. The stain was allowed to sit for 10 min. Afterwards, the stained pollen was observed under a microscope to remove any excess liquid. For SEM studies, anthers at the dehiscence stage were collected and examined using a SEM (JSM‐6610LV; JEOL, Japan).

For Safranin O/Fast green staining, the hypocotyls of 7‐day‐old seedlings were longitudinally cut, stained, and observed under a CX‐31 microscope (Olympus, Japan).

### Complementation assay

The *BnHL* coding sequences were amplified from *B. napus* cDNA and cloned into the pBI121‐GFP vector. The plasmid was transformed into the *Athl* mutant using the floral dip method, mediated by the *Agrobacterium tumefaciens* strain GV3101. Three independent transgenic lines were selected for hypocotyl length measurement and phenotypic analysis.

### Subcellular localization and *in situ* hybridization

The CDS of *BnHL* was inserted into the fusion expression vector pBI221‐enhanced green fluorescent protein (eGFP) plasmid. Protoplasts were prepared from young *Nicotiana benthamiana* leaves, and the empty eGFP and fusion expression vectors were transiently transfected into the protoplasts using the polyethylene glycol‐mediated transfection method. The protoplasts were then incubated in the dark for 16 h at 25 °C, and the localization of the *BnHL*‐eGFP fusion protein was determined by positive fluorescence microscopy (Leica microsystems, Germany).

Three hypocotyls from 7‐day‐old *B. napus* seedlings were fixed in 4% paraformaldehyde at 4 °C overnight. Subsequent procedures, including paraffin embedding, hybridization and detection, followed established protocols (Li *et al*., 2019). Sense and antisense RNA probes were generated by amplifying specific primers using SP6 and T7 polymerases respectively. Anti‐digoxigenin conjugated to alkaline phosphatase was used to detect digoxigenin‐labelled RNAs. Images were performed using a ECLIPSE CI microscope (Nikon, Tokyo, Japan).

### Sequence analysis

The gene structures of *AtHL* and *BnHL* were analysed using GSDS2.0 (http://gsds.gao‐lab.org/). Motif analysis of the amino acid sequences was performed using MEME (https://meme‐suite.org/meme/tools/meme). The three‐dimensional spatial structure of the proteins was predicted using SWISSMODEL (http://swissmodel.expasy.org/) and visualized using PyMOL (https://pymol.org/).

### CRISPR/Cas9 vector construction and plant transformation

A BLATP search identified other *BnHL* copies in the Westar genome (https://yanglab.hzau.edu.cn/) (Yang *et al*., 2023), including *BnHL‐a* (BnaC06T0380500WE), *BnHL‐b* (BnaA07T0303600WE) and *BnHL‐c* (BnaA07T0303500WE) (Figure S3). To knock out *BnHL*, two sgRNA targets were designed using CRISPR P2.0 (http://crispr.hzau.edu.cn/CRISPR2/; Liu *et al*., 2017). These sgRNA sequences were cloned into the pKSE401 CRISPR/Cas9 vector and transformed into Wes hypocotyls using *Agrobacterium tumefaciens* mediation (Cheng *et al*., 2021). Transgenic plants were screened and confirmed by antibiotic selection and PCR. The plants used for agronomic trait evaluation were planted in the Chengdu Plain, which is suitable for winter rapeseed growth. Four independent biological replicates for hl‐T_3_‐3, hl‐T_3_‐7 and Wes were selected for maturity analysis.

### DNA extraction and identification of transgenic plants

Leaves were collected from Wes and transgenic lines, and genomic DNA was extracted using the cetyltrimethylammonium bromide method (Doyle, 1990). Each plant was tested for transgenicity using PCR primers that amplified Cas9 fragments. All transgenic plants were verified by PCR and the PCR products of the target genes were verified by Sanger sequencing. The mutation types were identified by comparing the sequencing results with the reference sequences of the WT control.

### Chi‐squared test

Seeds obtained from self‐pollination using *hl‐3‐8* and *hl‐7‐12* were separately sown in the soil, resulting in the germination of 86 and 75 T_2_ generation seedlings respectively. Sequencing results and hypocotyl phenotypes were recorded and observed after 7 days of germination. A chi‐squared goodness‐of‐fit test was used to assess the expected 3:1 ratio. With a chi‐squared critical value of χ^2^_0.05,1_ = 3.84, the null hypothesis (H0) was accepted when χ^2^ < 3.84.

## Author contributions

RW, MW and JF conceived and designed the experiments. JF, MY, SL, ZX and MW contributed to the investigation. YZ and GC contributed to data analysis. JF, ZN, PL and RZ contributed to data curation. JF contributed to the writing, original draft preparation and visualization. RW contributed to the writing, reviewing, editing and funding acquisition. All the authors have read and agreed to the published version of the manuscript.

## Conflict of interest

The authors declare that they have no conflicts of interest.

## Supporting information


**Figure S1.** Phenotypes of the mutants during 1–4 weeks.
**Figure S2.** Identification of *Athl* mutant.
**Figure S3.** Genomic sequence alignment of three functional copies of *BnHL* in Westar.
**Figure S4.** Sequencing results for all editing mutants.
**Table S1.** *Arabidopsis* orthologues of candidate genes.
**Table S2.** Primers used in this study.
**Table S3.** Main agronomic traits of hl‐T_3_‐3 and hl‐T_3_‐7.

## Data Availability

The DNA sequences are available in the National Library for Biotechnology Information (NCBI) database under the Bioproject accession number PRJNA627442.
