# Supplementary material for: A visible seedling‐stage screening system for the Brassica napus hybrid breeding by a novel hypocotyl length‐regulated gene BnHL
Source: Plant Biotechnol J. 2024 Nov 6;23(2):442–53. doi: 10.1111/pbi.14507 (PMC11772316; doi:10.1111/pbi.14507)
Supplement: Supplementary file 1 — Figure S1. Phenotypes of the mutants during 1–4 weeks. Figure S2. Identification of Athl mutant. Figure S3. Genomic sequence alignment of three functional copies of BnHL in Westar. Figure S4. Sequencing results for all editing mutants. Table S1. Arabidopsis orthologues of candidate genes. Table S2. Primers used in this study. Table S3. Main agronomic traits of hl‐T3‐3 and hl‐T3‐7. [file PBI-23-442-s001.docx]

1. **Figure S1** Phenotypes of the mutants during 1-4 weeks.
2. (a) Planting diagram per pot. (b) The phenotypic comparison between the mutants and WT
3. plants was conducted at 1 week, 2 weeks, and 4 weeks of growth. (c) Hypocotyl phenotypes of
4. the mutants and WT under white light.


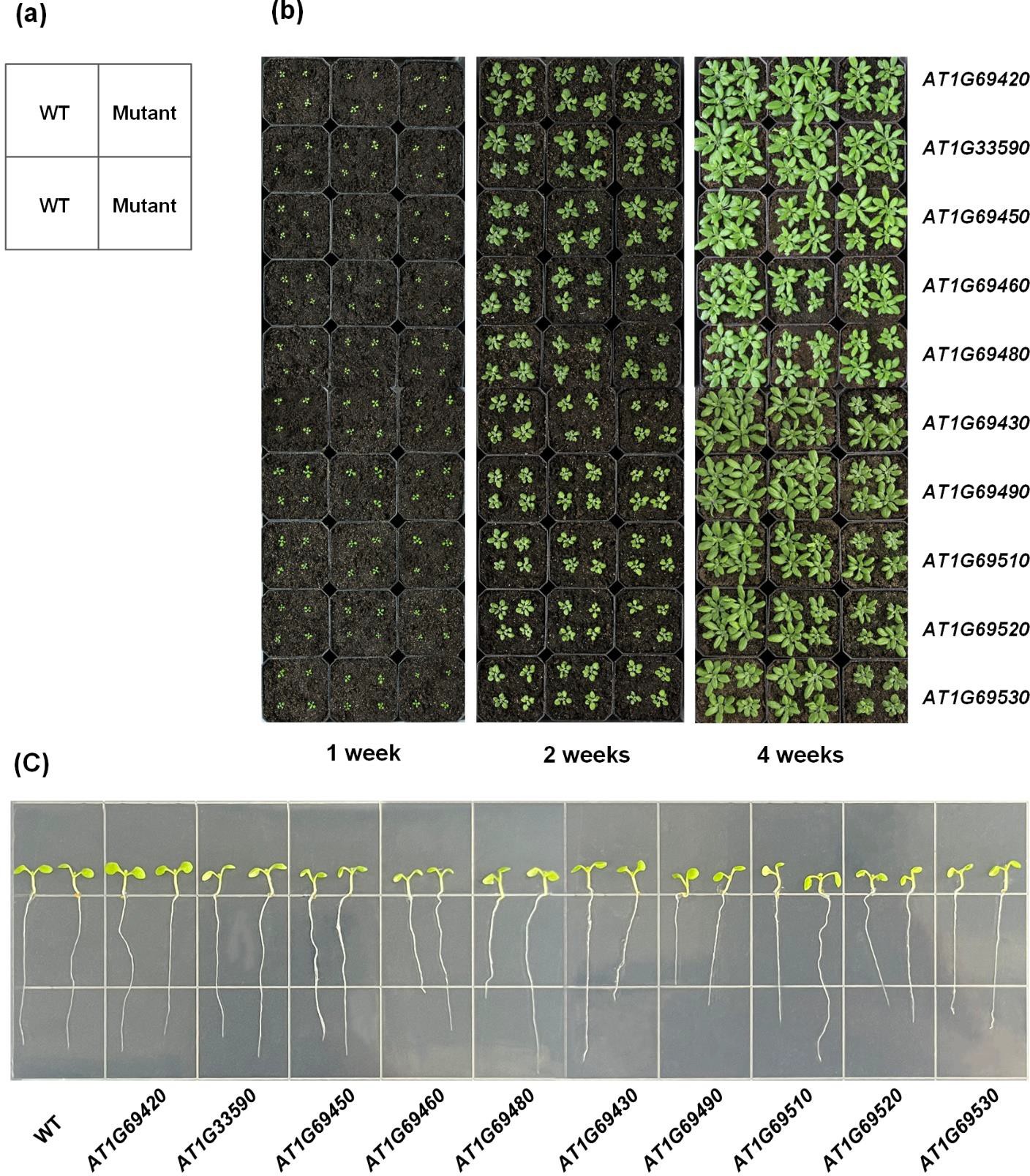


1. **Figure S2** Identification of *Athl* mutant.
2. M indicate DNA maker.


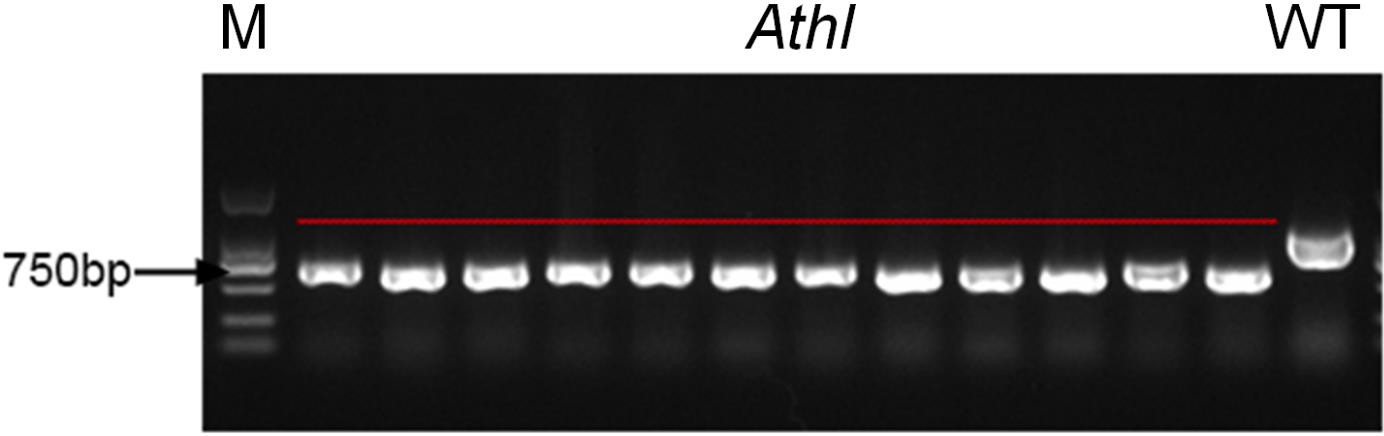


1. **Figure S3** Genomic sequence alignment of three functional copies of *BnHL* in Westar.
2. The PAM is underlined, red background indicates the exact same sequence, dark spots are used
3. to indicate the positions of gaps.


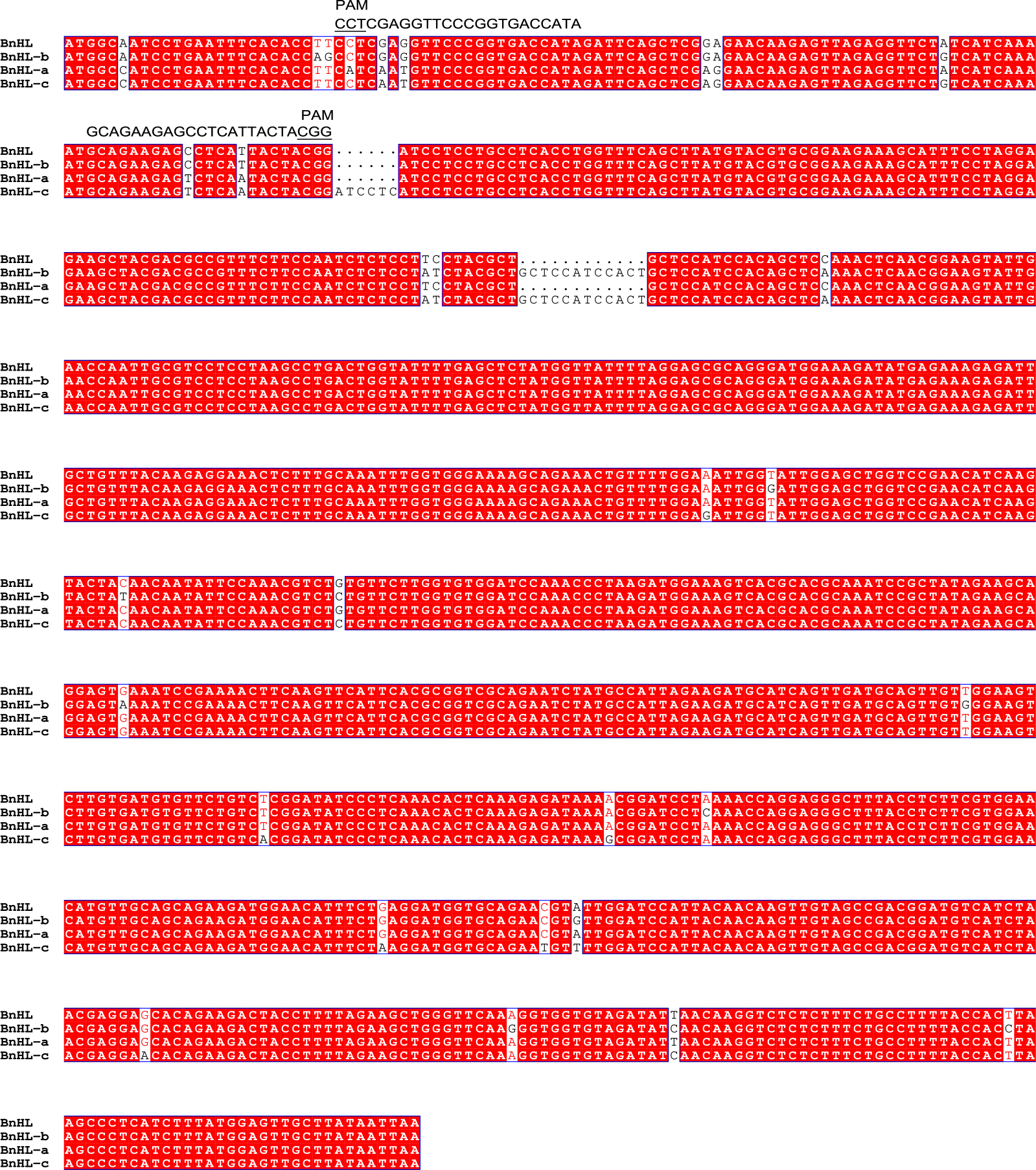


1. **Figure S4** Sequencing results for all editing mutants.
2.
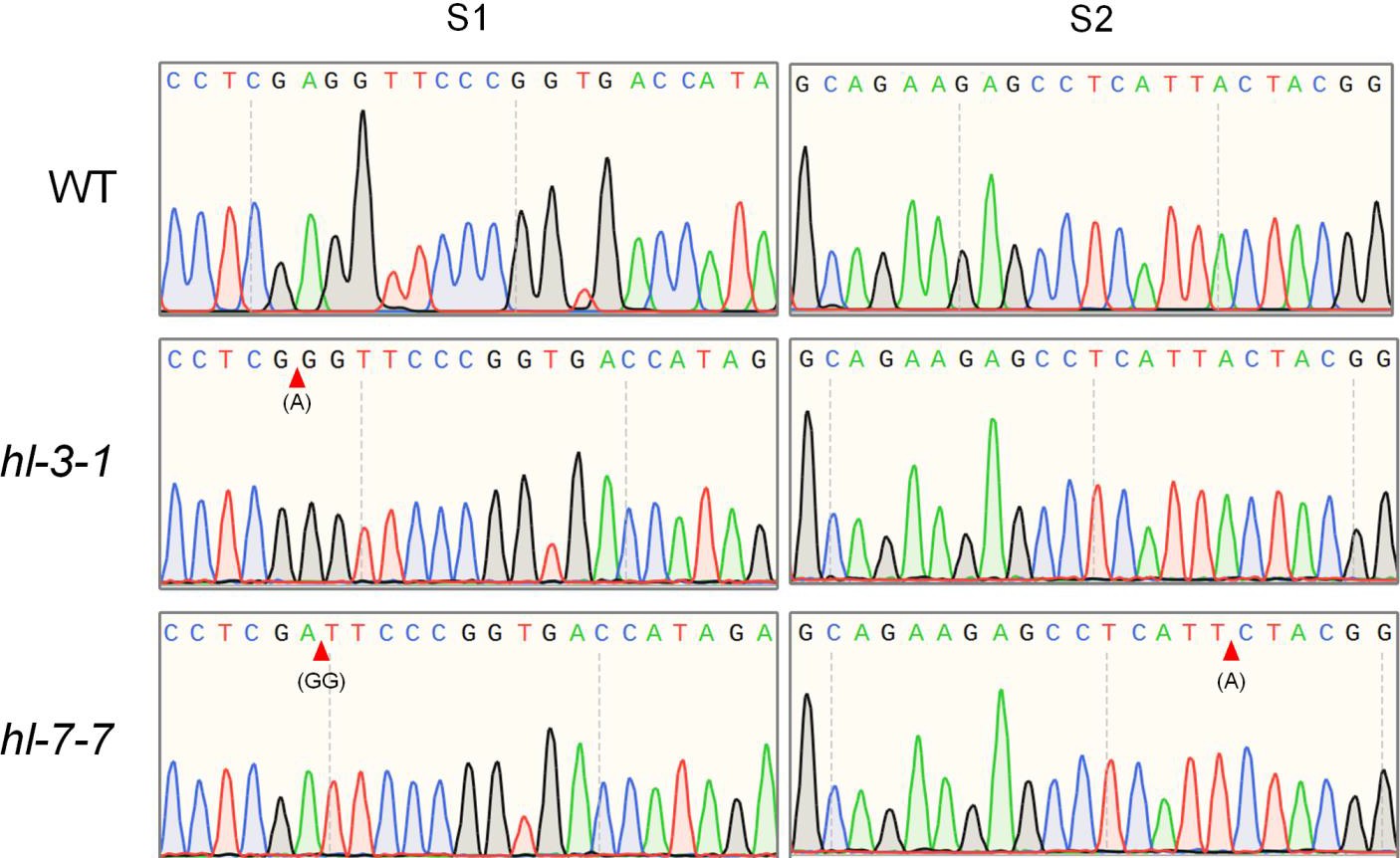
Red arrowheads indicate editing sites, the contents of the parentheses are the type of editing.
3. **Table S1** Arabidopsis orthologs of the candidate genes.

| Gene ID (*Brassica napus*) | *A. thaliana* ortholog | Coverage | Similarity |
| --- | --- | --- | --- |
| LOC106406361 | AT1G69420 | 100% | 90.07% |
| LOC106406259 | AT1G33590 | 100% | 83.92% |
| LOC106406116 | AT1G69450 | 99% | 87.08% |
| LOC106406671 | AT1G69460 | 99% | 84.98% |
| LOC106403036 | AT1G69480 | 98% | 86.77% |
| LOC106404487 | AT1G69430 | 100% | 83.75% |
| LOC106402938 | AT1G69490 | 100% | 82.42% |
| LOC106406884 | AT1G69510 | 100% | 73.86% |
| LOC106404716 | AT1G69520 | 99% | 79.83% |
| LOC106405564 | AT1G69523 | 100% | 73.75% |
| LOC106406215 | AT1G69530 | 100% | 92.77% |

1. **Table S2** Primers used in this study.

| Usage | primer | sequence (5'-3') |
| --- | --- | --- |
|  | 420-F | TGATTTGCTCTAATGCGTGTG |
|  | 420-R | TACTTTGGAGTGACGCAATCC |
|  | 590-F | GCTTTCAAAGCGGGTATAACC |
|  | 590-R | TTCCGACAGATCGATGAAATC |
|  | 450-F | GGGACTTGGCCACTTATTAGG |
|  | 450-R | CGAGTTCAAAATATGTCAAGTTTCC |
|  | 460-F | ATTCTCATGATCACTGCCTCC |
|  | 460-R | TAAAAGCTGGGCTAATGTTGC |
|  | 480-F | AAGCCTCAACTGTTCCTCTCC |
|  | 480-R | TTTTTCGTGCCCTGTATGATC |
| Arabidopsis mutant identification | 430-F | CTTGGGACACAATCTCTCCAG |
|  | 430-R | ACGCTAATCCCATATTTTGGG |
|  | 490-F | CTTTTTAACCGTGGCTGTTTG |
|  | 490-R | GTCCCCGAACCAACTAGACTC |
|  | 510-F | GTTTCGCCTGAAGAATAAGCC |
|  | 510-R | AATCTCCATTCTGTAACCGGC |
|  | 520-F | AATTTAAAGGACGATGGAGGC |
|  | 520-R | ACGAATGATGCAATCCAGTTC |
|  | 530-F | CAAAGCAGACCACTATGACCC |
|  | 530-R | TGTTCGGTAAGGCGTTGTTAG |
|  | AtHL-LP | CAAGGAGGGGTTTTCATCTTC |
|  | AtHL-RP | CTTGAAGTTGGGACCTGTTCC |
|  | LBb1.3 | ATTTTGCCGATTTCGGAAC |
| Cloning cDNA of  *BnHL* gene | BnHL-F | ATGGCAATCCTGAATTTCACAC |
|  | BnHL-R | TTAATTATAAGCAACTCCATAAAG |
|  | 121-HL-F | ttggagagaacacgggggactctagaATGGCAATCCTGAATTT CAC |
|  | 121-HL-R | tcctcgcccttgctcaccatggtaccATTATAAGCAACTCCATA  AAGATGAGGGC |
| Vector construction | 221-HL-F | acgggggactctagaggatccATGGCAATCCTGAATTTCAC  ACC |
|  | 221-HL-R | gctcaccataagcttgtcgacATTATAAGCAACTCCATAAA  GATGAGGG |
|  | 35S | GACGCACAATCCCACTATCC |
|  | EGFP-N-3 | CGTCGCCGTCCAGCTCGACCAG |
| *In situ* hybridization | Hy-1 | CCAGGTGAGGCAGGAGGATCCGTA |
|  | Hy-2 | TTTGGAGCTGTGGATGGAGCAGCG |

|  | Hy-3 | GATGTTCGGACCAGCTCCAATACC |
| --- | --- | --- |
|  | Hy-4 | GGCATAGATTCTGCGACCGCGTGA |
|  | Hy-5 | CCAATACGTTCTGCACCATCCTCA |
|  | hpt557-F | ACACTACATGGCGTGATTTCAT |
|  | hpt557-R | TCCACTATCGGCGAGTACTTCT |
| *BnHL* editing test | 23KN57-F | ggagtgagtacggtgtgcGAGAAATAGCAATGGCAATCC TG |
|  | 23KN57-R | gagttggatgctggatggGCTTTCTTCCGCACGTACAT |
|  | BnHL-F | CCTAGGAGGAGCTACGACG |
| RT-qPCR | BnHL-R | GTTTCCTCTTGTAAACAGCAATCTC |
|  | BnActin-F | TCTTCCTCACGCTATCCTCC |
|  | BnActin-R | AGCCGTCTCCAGCTCTTG |

14

**Table S3** Main agronomic traits of hl-T_3_-3 and hl-T_3_-7.

| Plants | 1000-seed weight(g) | Plant height (cm) | Silique number per  plant | Number of seeds per  silique | Yield per plant (g) |
| --- | --- | --- | --- | --- | --- |
| Wes | 3.40±0.12 | 174.11±5.16 | 338.04±8.36 | 18.37±0.31 | 19.66±0.58 |
| hl-T_3_-3 | 3.31±0.11 | 176.49±3.38 | 305.50±12.79* | 18.21±0.49 | 17.72±0.50 |
| hl-T_3_-7 | 3.30±0.12 | 168.99±4.11 | 321.64±6.51 | 17.49±0.32 | 17.66±0.63 |

Note: The data represents the mean ± S E; statistical analysis was performed by t-test. T_3_, transgenic generation 3.
